# Hyper-Reflecting Foci in Multiple Sclerosis Retina Associate With Macrophage/Microglia-Derived Cytokines in Cerebrospinal Fluid

**DOI:** 10.3389/fimmu.2022.852183

**Published:** 2022-05-19

**Authors:** Marco Puthenparampil, Tommaso Torresin, Silvia Franciotta, Annachiara Marin, Federica De Napoli, Valentina Annamaria Mauceri, Silvia Miante, Elisabetta Pilotto, Edoardo Midena, Paolo Gallo

**Affiliations:** ^1^ Multiple Sclerosis Centre, Neurology Clinic, Department of Neuroscience, Università degli Studi di Padova, Padova, Italy; ^2^ Ophthalmology Clinic, Department of Neuroscience, Università degli Studi di Padova, Padova, Italy

**Keywords:** multiple sclerosis, OCT, microglia, retina, cerebrospinal fluid, hyper-reflecting foci

## Abstract

**Background:**

Increasing evidence suggests that retinal hyper-reflecting foci (HRF) might be clusters of activated and proliferating microglia. Since microglia are widespread activated in multiple sclerosis (MS) brain, its evaluation in retina may help to understand and monitor MS-related pathology.

**Aim:**

This study aims at investigating the association of HRF with cerebrospinal fluid (CSF) cytokines and MRI parameters in relapsing–remitting MS (RRMS).

**Methods:**

Nineteen RRMS at clinical onset and 15 non-inflammatory neurological disorders (NIND) underwent brain 3T MRI and CSF examination. Optical coherence tomography (OCT) analysis, including HRF count, was performed on RRMS patients. Sixty-nine cytokines/chemokines were analyzed in the CSF by multiplex technology.

**Results:**

In RRMS, HRF count in the ganglion cell layer (GCL) was associated with IL-1Ra, IL-9, IL-15, IFN-γ, and G-CSF. Moreover, in RRMS patients CSF concentrations of IL-1Ra and G-CSF associated with global cortical thickness. The HRF count in the inner nuclear layer (INL) correlated with IL-22, IL-34, IL-35, CXCL-2, CXCL-10, and CXCL-13, and multivariate analysis confirmed a strong association (r^2^: 0.47) with both CXCL-2 (β: -0.965, p = 0.0052) and CXCL-13 (β: 0.241, p = 0.018). This latter cytokine increased in RRMS with high HRF count compared with NIND and RRMS with low HRF count. Finally, the CXCL-13/CXCL-2 ratio strongly associated with HRF count (r: 0.8, p < 0.005) and cortical lesion volume (r: 0.5, p < 0.05).

**Conclusions:**

The association of HRF with intrathecally produced monocyte/microglia-derived cytokines confirms their microglial origin and indicates they are worth further evaluating as markers of activated microglia.

## Introduction

Retinal hyper-reflecting foci (HRF) have been observed by optical coherence tomography (OCT) in a wide range of neurological and ophthalmological diseases (1–4). However, their origin is still debated, and two main hypotheses have been advocated. The first hypothesis suggests that they represent extravasated lipoproteins. Indeed, in Fabry disease (5) HRF count was found to correlate with both globotriaosylsphingosine serum concentration and vessel tortuosity, supporting shared pathogenic mechanism(s). Since both the retina and macula are highly vascularized, it has been hypothesized that blood–retina barrier dysfunction might facilitate concomitant endothelial glycosphingolipid deposition, resulting in a pathological hyperreflectivity of the capillary plexus in the inner retina. The second hypothesis suggests that HRF, characterized by well-defined morphological features, are composed of clusters of activated and proliferating microglia. As a matter of fact, HRF have been observed in pathologies not associated with retinal lipid deposition. Moreover, in relapsing–remitting multiple sclerosis (RRMS) HRF are increased compared to healthy controls (3) and associated with MRI parameters of cortical inflammation (6). Finally, the reported association between HRF and inflammatory markers (i.e., IL-8, V-CAM-1) in aqueous humor in patients with intractable macular edema also speaks in favor of the microglia-origin hypothesis (7).

Interestingly, beside advanced neuroimaging methodologies that have been tested for the *in vivo* evaluation of activated microglia in the brain, OCT with a single linear scan through the macula proved to identify and correlate the HRF with different parameters of local inflammation and damage (3, 5, 7–10). Moreover, a strong correlation of HRF with cortical lesion load and clinical and radiological disease activity have been observed in MS (3, 6).

To further explore the origin of HRF, we designed a cross-sectional study in RRMS patients at clinical onset and untreated, aiming at evaluating the possible correlation of HRF count with cerebrospinal fluid (CSF) cytokines/chemokines and magnetic resonance imaging (MRI) parameters of both gray and white matter inflammation and degeneration.

## Materials and Methods

### Study Design and Participants

Subjects with a diagnosis of RRMS achieved at the Multiple Sclerosis Centre, University Hospital of Padua, between January 2014 and June 2020 were recruited in this cross-sectional, single-center study. All subjects underwent neurological evaluation and Expanded Disability Status Examination (EDSS), CSF analysis, OCT, and brain and spinal cord MRI at the time of the diagnosis. Inclusion criteria were 1) diagnosis of RRMS achieved according to the revised 2017 McDonald criteria (11); 2) interval between clinical onset and diagnosis <18 months; and 3) age between 18 and 60 years. Exclusion criteria were 1) systemic and ophthalmologic disorders (i.d. diabetes); 2) diagnosis of progressive MS; 3) steroid therapy in the month prior to OCT acquisition; 4) previous clinical history of optic neuritis; 5) evidence of subclinical optic neuritis (inter-eye difference in peripapillary RNFL of >20% and optic nerve hyperintensity in ≥2 slices at Brain MRI Double Inversion Recovery Sequence); and 6) no steroid therapy in the previous 28 days and no history of immunomodulating or immunosuppressive therapy before lumbar puncture. Disease duration was defined as the interval between the first clinical symptom attributable to MS and the date of the OCT evaluation coinciding with the diagnosis. The 19 RRMS patients included were compared with a group of 15 patients with a final diagnosis of Non-Inflammatory Neurological Disease (NIND). The NIND group was constituted by subjects complaining tension headache, transient subjective sensory symptoms, and psychosomatic disorders, as well as unspecific white matter alterations who underwent a detailed diagnostic workup including routine blood tests; B12 vitamin, folate, and angiotensin-converting enzyme (ACE) concentration as well as immunological screening (detecting ANA, ANCA, ENA, anti-dsDNA, anti-β_2-_glycoprotein I, anti-cardiolipin, and LAC); CSF examination; and brain and spinal cord MRI to exclude neurological disorders, as previously described (12). Even if no evidence of neurological or systemic diseases was achieved in these subjects, these patients were classified as NIND rather than healthy controls.

No difference in demographic and clinical variables was observed between NIND and RRMS (**Table 1**). The study was conducted in agreement with the Declaration of Helsinki and approved by the local Ethic Committee (Comitato Etico per la Sperimentazione Clinica dell’Azienda Ospedaliera di Padova, prot. n. 17760).

**Table 1:** Clinical and demographic characteristics of patients.

	NIND (15)	RRMS (19)
Gender ratio (F/M)	2.0 (10/5)	1.7 (12/7)
Age at CSF (y)	43.9 ± 8.1	34.6 ± 9.9
Disease duration at CSF (m)	n.a.	3.5 ± 4.0
Disease duration at OCT (m)	n.a.	4.1 ± 4.0
EDSS	n.a.	2.0 (1.0-3.0)

NIND, other non-inflammatory neurological diseases; RRMS, relapsing–remitting multiple sclerosis; y, years; m, months; EDSS, Expanded Disability Status Scale. CSF, cerebrospinal fluid; OCT, optical coherence tomography; n.a., not applicable.

### CSF Cytokine Investigation

CSF specimens were collected by non-traumatic lumbar puncture between 8.00 and 9.00 a.m., as previously reported (13). Following routine examination (consisting in cell count and differentiation, calculation of albumin ratio and quantitative IgG indexes, detection of oligoclonal IgG bands), paired serum and CSF specimens were stored in 0.5-ml aliquots at -80°C until further analysis. The CSF concentration of 69 cytokines was assessed by multiplex technology (Bio-Plex Pro Human Cytokine, GF and Diabetes 27-Plex Panel, Bio-Plex Pro Human Chemokines 40-Plex Panel, Bio-Plex Pro Human Inflammation Assays 37-Plex Panel) as already described (12). Briefly, for each molecule the percentage of detectable concentration was evaluated. Cytokines not detected in all (MS and NIND) samples were excluded from the analysis. When the same cytokine was detectable by two kits, results from the kit with higher sensitivity were further analyzed.

### Spectral Domain OCT and Hyper-Reflective Foci Count

All MS patients underwent spectral domain OCT (Spectralis; Heidelberg Engineering, Carlsbad, CA; Heidelberg Eye Explorer version 1.7.0.0) examination of both eyes, in a dark room without the injection of any mydriatic agent. In line with recent publications (3, 6, 14), the analysis of the central linear scan of the macular map, crossing the fovea, was considered for HRF counting. HRF were counted in the area included between two perpendicular lines to Brunch’s membrane traced at 1,500 μm both temporally and nasally from the center of the fovea. HRF were defined as isolated, small-size (<30 μm), punctiform elements with moderate reflectivity (similar to that of the nerve fiber layer) but without any back shadowing. The count was performed in GCIP and INL separately. The presence of HRF was rated by two independent blind observers (PM, TT). For each patient, HRF count was expressed as the median between the 2 eyes, because a significant correlation was demonstrated between the two eyes for both GCL and INL HRF count (**Supplementary Figure 1**). All examinations were checked for sufficient quality using the OSCAR-IB criteria (15). The results are reported in accordance with the Advised Protocol for OCT Study Terminology and Elements (APOSTEL) (16, 17). Since the signal strength in macular scan of 2 eyes from 2 MS patients was not optimal (<15), these scans were not analyzed.

### MRI Data Acquisition

MRI was achieved on a 3.0-T scanner (Ingenia, Philips Medical Systems, Best, The Netherlands) with 33-mT/m power gradient and a 32-channel head coil. No major hardware upgrades occurred during the study, and bimonthly quality assurance sessions were done for measurement stability. The MRI protocol included the following sequences: (i) three-dimensional (3D) T1 MPRAGE: repetition time (RT) = 7.8 ms, echo time (ET) = 3.6 ms; 180 contiguous axial slices with the off-center positioned on zero with thickness of 1.0 mm; flip angle = 8°; matrix size = 220 × 220; FOV 220 × 220 × 180 mm^3^; (ii) 3D-FLAIR: RT = 4,800 ms, ET = 310 ms, inversion time (IT = 1,650 ms; 365 contiguous axial slices with thickness of 1.0 mm; matrix size 256 × 256; and FOV = 256 × 256 × 182 mm^3^; and (iii) 3D-DIR: RT= 55,000 ms, ET = 284 ms, inversion time (IT) = 2,550 ms; contiguous axial slices with thickness of 1.0 mm; matrix size 212 × 212; and FOV = 256 × 256 × 182 mm^3^.

### MRI Data Processing

T1-weighted images were processed following these procedures: correction for magnetic field inhomogeneity, performed with ANTs N4 Bias Field Correction tool, (18) brain extraction, performed with ants Brain Extraction tool, (19) brain segmentation, performed with FSL fast(20) tool and lesion filling, performed with FSL lesion filling tool (21). The FLAIR images were processed following these steps: correction for magnetic field inhomogeneity, performed with ANTs N4BiasFieldCorrection (18) tool and brain extraction, performed with FSL bet tool (22). The processed FLAIR image was then registered to the processed T1-weighted image by using ants Registration SyN Quick (19) tool and the estimated transformation was applied to the WM lesion mask. DIR images were corrected for magnetic field inhomogeneity with ANTs N4BiasFieldCorrection (18) tool and was then registered to the processed T1-weighted image by using FSL flirt tool (23, 24). Thus, the GM lesion mask, designed in the DIR space, was registered to the same space applying the transformation estimated in the previous step. In order to evaluate the role of the WM lesions on the optic radiations (ORs), we used a previously described atlas, (25) which includes the tracts of the OR, defined in the MNI152 space. To conduct a subject-level analysis, MNI152 brain image was registered to the processed T1-weighted image as described elsewhere. After having applied the estimated transformations to the OR tracts, an expert neuroradiologist fixed the lower thresholds for the left OR tract to 0.7 and for the right OR to 0.55. Then, we quantified global and normalized WM and GM lesion volume (WMLV and GMLV) as well as WM lesion volume and their normalized values in both ORs.

### Statistical Analysis

Data were reported as mean (± standard deviation) or median (range) for continuous variables. Group differences between RRMS patients and NIND were tested by the chi-square test for sex and by the T-test for age. Non-parametric Spearman correlation was applied to correlate HRF count and cytokines. Multiple linear regression analysis considered HRF count as a dependent variable and cytokines as an independent variable. Differences between ONIND and RRMS subgroups were tested by the Kruskal–Wallis test corrected with multiple-comparison Dunn’s test. A p-value lower than 0.05 was considered statistically significant. Prism 9.2.0 was used for all the analyses.

## Results

### GCL and INL HRF Counts Associated With Different Patterns of Monocyte-Derived CSF Cytokines

**Figure 1:**
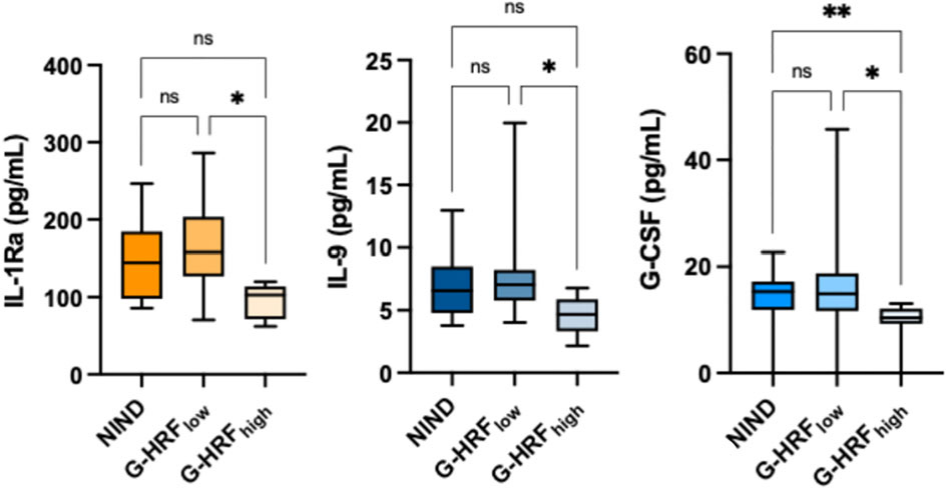
GCL HRF count associates with monocyte-derived CSF cytokines. ONIND, other non-inflammatory neurological diseases; G-HRF_low_, RRMS patients with GCL HRF count ≤ 8.5; G-HRF_high_, RRMS patients with GCL HRF count > 8.5; *p < 0.05; **p < 0.01. n.s., not significant.

GCL HRF count associated with IL-1Ra (r: -0.67, p = 0.0016), IL-9 (r: -0.61, p = 0.0056), IL-15 (r: -0.63, p = 0.0041), G-CSF (r: -0.51, p = 0.0257), and IFN-γ (r: -0.49, p = 0.0334) (**Table 2**, **Supplementary Table 1**). On the basis of GCL HRF median count (i.e., 8.5), patients were divided into high GCP HRF count (G-HRF_high_) and low GCP HRF count (G-HRF_low_). Interestingly, significantly lower levels of IL-1Ra, IL-9, and G-CSF were found in G-HRF_high_ compared to G-HRF_low_ (**Figure 1**). Finally, IL-1Ra, IL-9, and G-CSF were reciprocally correlated (p < 0.0005 for all correlations).

**Table 2:** CSF concentrations of cytokines correlating with GCL HRF count.

	ONIND	G-HRF_low_	G-HRF_high_	ONIND vs. G-HRF_low_ (p-value)^#^	ONIND vs. G-HRF_high_ (p-value)^#^	G-HRF_low_ vs. G-HRF_high_ (p-value)^#^
Disease duration at CSF (m)	n.a.	3.3 ± 3.9	3.7 ± 4.3	n.a.	n.a.	0.6
EDSS	n.a.	2.0 (1.0-2.5)	2.0 (1.0-3.0)	n.a.	n.a.	0.3
Brain MRI gad+ (%)	n.a.	40.0%	37.5%	n.a.	n.a.	1.0
Spinal cord MRI gad+ (%)	n.a.	30.0%	12.5%	n.a.	n.a.	1.0
Radiological activity (%)	n.a.	50.0%	37.5%	n.a.	n.a.	1.0
Clinical activity (%)	n.a.	60.0%	37.5%	n.a.	n.a.	0.7
Disease activity (%)	n.a.	80.0%	62.5%	n.a.	n.a.	1.0
IL1-Ra (pg/mL)	147.5 ± 51.8	164.1 ± 61.4	95.1 ± 21.8	>0.999	0.0681	0.0244
IL-9 (pg/mL)	7.0 ± 2.6	8.0 ± 4.4	4.6 ± 1.5	>0.999	0.0789	0.0281
IL-15 (pg/mL)	32.4 ± 14.7	34.2 ± 12.5	20.6 ± 9.4	>0.999	0.1187	0.0946
G-CSF (pg/mL)	14.6 ± 5.3	16.8 ± 11.6	9.6 ± 3.9	>0.999	0.0095	0.0249
IFN-γ (pg/mL)	2.9 ± 1.2	2.8 ± 1.0	2.2 ± 0.6	>0.999	0.4621	0.5566

NIND, other not inflammatory neurological diseases; G-HRF_low_, RRMS patients with GCL HRF count ≤8.5; G-HRF_high_, RRMS patients with GCL HRF count >8.5; ^#^p-values from Kruskal-Wallis test corrected with multiple comparison Dunn’s test; n.a., not applicable.

**Figure 2:**
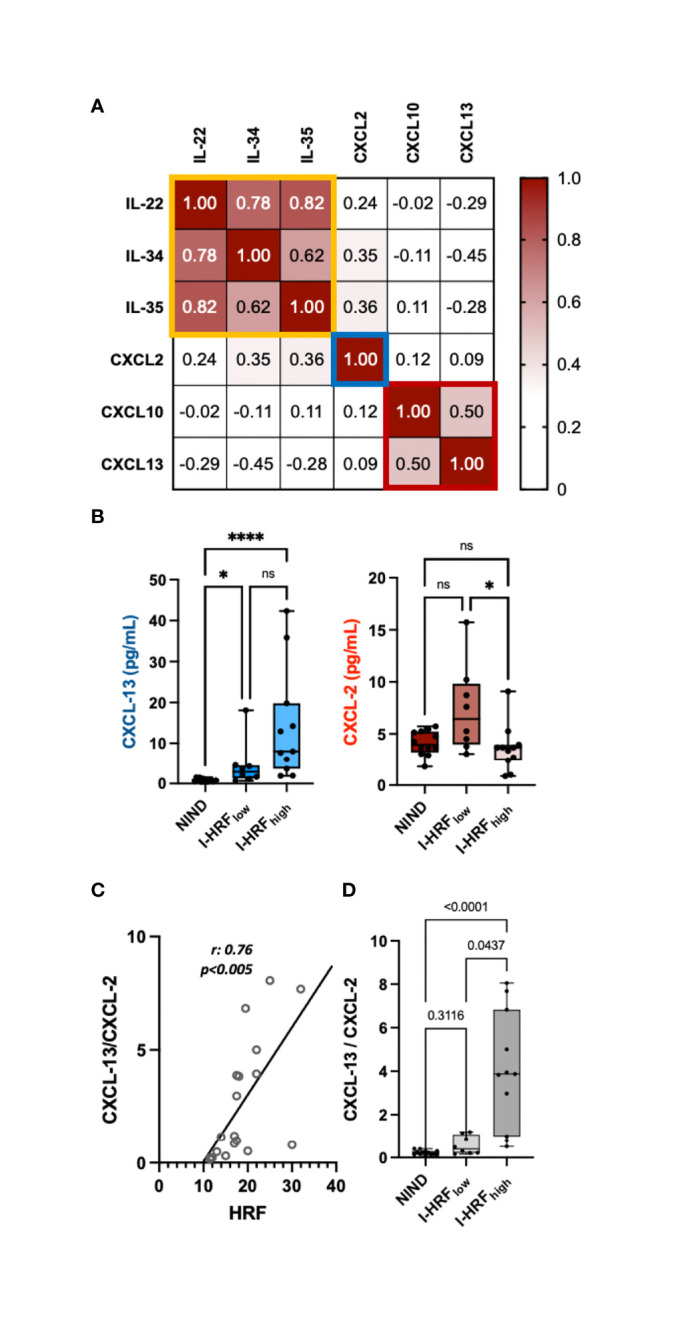
INL HRF count associates with an imbalance between CSF concentrations of CXCL-13 and CXCL-2. **(A)** Correlation matrix revealed 3 clusters of cytokines, narrowed to 2 by multiple regression analysis (CXCL-13 and CXCL-2). **(B)** While CXCL-13 CSF concentrations progressively increased from ONIND to both I-HRF_low_ and I-HRF_high_, CXCL-2 significantly decreased between RI-HRF_low_ and I-HRF_high_. **(C)** The ratio between CXCL-13 and CXCL-2 strongly associated with HRF count in INL; **(D)** this ratio significantly increased in I-HRF_high_ compared with both ONIND and I-HRF_low_. ONIND, other non-inflammatory neurological diseases; I-HRF_low_, RRMS patients with GCL HRF count ≤ 17.5; I-HRF_high_, RRMS patients with GCL HRF count > 17.5; *p < 0.05; ; **** p < 0.001. n.s., not significant.

INL HRF count associated inversely with IL-22 (r: -0.47, p = 0.043), IL-34 (r: -0.51, p = 0.027), IL-35 (r: -0.47, p = 0.044), and CXCL-2 (r: -0.53, p = 0.020) and directly with CXCL-10 (r: 0.47, p = 0.042) and CXCL-13 (r: 0.50, p = 0.03) (**Table 3** and **Supplementary Table 2**). The correlation matrix revealed 3 clusters of cytokines, from whom IL-22, CXCL-2, and CXCL-13 were selected (**Figure 2A**). The regression model indicated that CXCL-2 associated inversely (β: -0.965, p = 0.0052) while CXCL-13 associated directly (β: 0.241, p = 0.018) with HRF count (r^2^: 0.47) (**Figures 2B, C**). Consequently, the CXCL-13/CXCL-2 ratio strongly associated with INL HRF count (r: 0.76, p = 0.0002) (**Figure 2C**). Patients were divided on the basis of median INL HRF count (i.e., 17.5), in INL HRF_high_ (I-HRF_high_) and INL HRF_low_ (I-HRF_low_). I-HRF_high_ had a CXCL-13/CXCL-2 ratio (4.05 ± 2.67) than both I-HRF_low_ (0.57 ± 0.43, p = 0.0001) and NIND (0.22 ± 0.11, p < 0.0001) (**Figure 2D**).

**Table 3:** CSF concentrations of cytokines correlating with INL HRF count.

	NIND	I-HRF_low_	I-HRF_high_	NIND vs. I-HRF_low_ (p-value)*^a^*	NIND vs. I-HRF_high_ (p-value)*^a^*	I-HRF_low_ vs. I-HRF_high_ (p-value)*^a^*
Disease duration at CSF (m)	n.a.	4.9 ± 2.5	2.5 ± ± 3.1	n.a.	n.a.	0.4
EDSS	n.a.	1.5 (1.0-3.0)	2.5 (1.5-2.5)	n.a.	n.a.	0.1
Brain MRI gad+ (%)	n.a.	25.0%	54.6%	n.a.	n.a.	0.6
Spinal cord MRI gad+ (%)	n.a.	25.0%	18.2%	n.a.	n.a.	1.0
Radiological activity (%)	n.a.	37.5%	45.5%	n.a.	n.a.	1.0
Clinical activity (%)	n.a.	37.5%	63.6%	n.a.	n.a.	0.4
Disease activity (%)	n.a.	62.5%	81.8%	n.a.	n.a.	0.6
CXCL-2 (pg/mL)	4.1 ± 1.2	7.4 ± 4.2	3.6 ± 2.2	0.2569	0.8726	0.0251
CXCL-13 (pg/mL)	0.8 ± 0.4	4.5 ± 5.7	14.0 ± 13.6	0.0217	<0.0001	0.4280

ONIND, other not inflammatory neurological diseases; I-HRF_low_, RRMS patients with INL HRF count ≤17.5; I-HRF_high_, RRMS patients with INL HRF count >17.5.

^a^p-values from the Kruskal–Wallis test corrected with multiple-comparison Dunn’s test; n.a., not applicable.

### CSF Cytokines Reflected Different Aspects of Cortical Pathology

While IL-1Ra and G-CSF concentrations correlated with global cortical thickness (r: 0.52, p = 0.022, and r: 0.49, p = 0.034 respectively), the CXCL-13/CXCL-2 ratio correlated with gray matter lesion volume (GMLV) (r: 0.465, p = 0.045).

## Discussion

The impossibility of obtaining histological specimens of the human retina *in vivo* keeps the question on the origin and pathologic significance of HRF still open. Nevertheless, indirect evidence, accumulated over the last decade, supports the hypothesis that these nodules are constituted by clusters of activated retinal microglia that migrate close to the blood-retinal barrier probably in response to detrimental triggers.

Inasmuch as a widespread microglial activation has been demonstrated by histological and neuroimaging (advanced MRI and PET techniques) studies in MS (26), this disease may constitute an ideal pathological field, where the origin of HRF is clarified and microglial behavior during the course of CNS inflammatory disorders is analyzed. In a previous study, we found that INL HRF count correlated with both inflammatory cortical pathology and INL thickness, suggesting HRF parallel gray matter rather than white matter damage in RRMS (6). These findings were in line with histological observations supporting a pivotal role for microglia and a marginal role for T cells in both retinal and cortical pathology in MS (27).

Thus, we investigated the possible correlation of HRF with a wide range of inflammatory soluble molecules (including monocyte-, microglia-, granulocyte-, lymphocyte-related cytokines) in the CSF of RRMS patients at clinical onset and untreated. The most significant and interesting finding was the inverse correlation between GCL HRF counts and G-CSF values. This cytokine showed a neuroprotective effect in a rat model of anterior ischemic optic neuropathy (rAION model) (28) and has been used to modulate several ON/retina animal models, including ON axotomy or crush injury, light-induced retinal damage, retinal ischemia and reperfusion, and oxygen-induced retinopathy (29–31). Because of the protective effect of IL-1Ra on the retina (32), the inverse association of G-CSF and IL-1Ra with microglial proliferation sounds plausible and may indicate a loss of protective effect by local microglia, suggesting a shift to a different phenotype.

Among several monocyte/microglia-derived cytokines/chemokines, CXCL-13, whose role in MS has been repeatedly ascribed (33, 34), seems particularly linked to INL HRF count. Our study further confirms that this cytokine is increased in MS CSF (33) and associated with cortical pathology (34). INL pathological changes appear particularly relevant in this context. Indeed, INL thickness has been linked to both an impairment of Müller cell-maintained retinal fluid homoeostasis and an increased blood–retina barrier permeability induced by the local production of pro-inflammatory cytokines (IL-1 and IL-6) and iNOS by microglia (35). Since RRMS patients with clinical or subclinical optic neuritis were excluded from this study, the increased HRF count assumes an unexpected and novel pathological significance, indicating that the retina might be an independent site of CNS involvement in MS and local microglia could activate in the absence of optic nerve or radiation inflammation.

Taken altogether, our data strongly support the hypothesis that HRF might be constituted, at least partially, by activated microglia. HRF should be interpreted in the context of the widespread microglial activation and proliferation that take place in MS CNS. The different concentrations of macrophage/microglia-derived cytokines/chemokines strongly indicate that these pro-inflammatory factors may also be produced in the retina, thus explaining the morphological changes observed here. Indeed, microglial activation in GCP and INL could induce a cytokine release that leads to BRB dysfunction and increases permeability determining an increase in the INL volume. This immunopathological hypothesis agrees with a recent observation on blood–brain barrier dysfunction associated with specific patterns of microglia-derived pro-inflammatory cytokines in the CSF (e.g., chitinase 3-like 1) (36). The microglial origin can also explain the correlation between Lyso-Gb3 and HRF count and vessel tortuosity in Fabry’s disease. Indeed, endothelial glycosphingolipid deposition may stimulate the recruitment and activation of local microglia.

The different patterns of cytokines/chemokines associated with HRF count are worth commenting. Indeed, I-HRF_high_ had higher CSF CXCL-13 than NIND and I-HRF_low_, while the CXCL-2 concentration was slightly increased in I-HRF_low_ and then dropped in I-HRF_high_. This behavior may reflect phenotypical changes of retinal microglia. Indeed, I- or G-HRF_low_ patients have CSF cytokine concentrations similar to those observed in NIND, suggesting a “physiological” low-level release of soluble mediators by microglia, in line with the homeostatic role of these cells. Furthermore, the association of increased HRF count with different cytokine profiles in I- or G-HRF_high_ suggests a microglial shift from a homeostatic to an activated profile, characterized by proliferation and production of higher levels of pro-inflammatory mediators. However, since no histological or cytological evaluation is here provided and considering that these data derive from a limited cohort of MS patients, our findings need to be handled with caution and need to be further confirmed. Longitudinal studies will reveal whether the association of CSF cytokines with HRF counts is worthy of further experimental development and will help to better weight our preliminary findings. Moreover, to further explore the origin of HRF, studies evaluating the parallel behavior of retinal and brain microglia should be performed [as mentioned above, PET allowed the observation of a widespread microglial activation in the gray matter of a small cohort of MS patients (37)].

In conclusion, our study links HRF count with soluble markers of microglial origin in CSF and MRI parameters of cortical pathology, further supporting the hypothesis that HRF are clusters of activated and proliferating microglia. Retinal HRF appear promising candidate biomarkers for elucidating *in vivo* the mechanisms behind microglial activation and proliferation in inflammatory and neurodegenerative brain disorders.

## Data Availability Statement

The raw data supporting the conclusions of this article will be made available by the authors, without undue reservation, upon reasonable request.

## Ethics Statement

The studies involving human participants were reviewed and approved by Comitato Etico per la Sperimentazione Clinica dell’Azienda Ospedaliera di Padova (Comitato Etico per la Sperimentazione Clinica dell’Azienda Ospedaliera di Padova, prot. n. 17760). The patients/participants provided their written informed consent to participate in this study.

## Author Contributions

MP: study concept and design, collection of clinical, OCT, and immunological data, writing and critical revision of the manuscript, statistical analysis of the data. TT: collection of OCT data, writing and critical revision of the manuscript. SF collection of clinical and immunological data, writing and critical revision of the manuscript. AM: collection of immunological data, writing and critical revision of the manuscript. FN: collection of clinical and immunological data. VM: collection of clinical and OCT data. MS: study concept and design, collection of clinical and OCT data. EP: study concept and design, writing and critical revision of the manuscript. EM: study concept and design, writing and critical revision of the manuscript. PG: study concept and design, writing and critical revision of the manuscript. All authors contributed to the article and approved the submitted version.

## Funding

This project was funded by “Progetto di Eccellenza 2020”, Department of Neuroscience, Università degli Studi di Padova, and “Roche per la Ricerca 2016”.

## Conflict of Interest

The authors declare that the research was conducted in the absence of any commercial or financial relationships that could be construed as a potential conflict of interest.

## Publisher’s Note

All claims expressed in this article are solely those of the authors and do not necessarily represent those of their affiliated organizations, or those of the publisher, the editors and the reviewers. Any product that may be evaluated in this article, or claim that may be made by its manufacturer, is not guaranteed or endorsed by the publisher.

## Supplementary Material

Supplementary Figure 1 Spearman Correlation of GCL HRF count between the two eyes **(A)** and of INL HRF count between the two eyes **(B)**.

Supplementary Table 1 Spearman Correlation between GCL HRF count and CSF cytokine concentrations in RRMS.

Supplementary Table 2 Spearman Correlation between INL HRF count and CSF cytokine concentrations in RRMS.
