# Supplementary figures and images for: Hyper-Reflecting Foci in Multiple Sclerosis Retina Associate With Macrophage/Microglia-Derived Cytokines in Cerebrospinal Fluid
Source: Front Immunol. 2022 May 19;13:852183. doi: 10.3389/fimmu.2022.852183 (PMC9160385; doi:10.3389/fimmu.2022.852183)

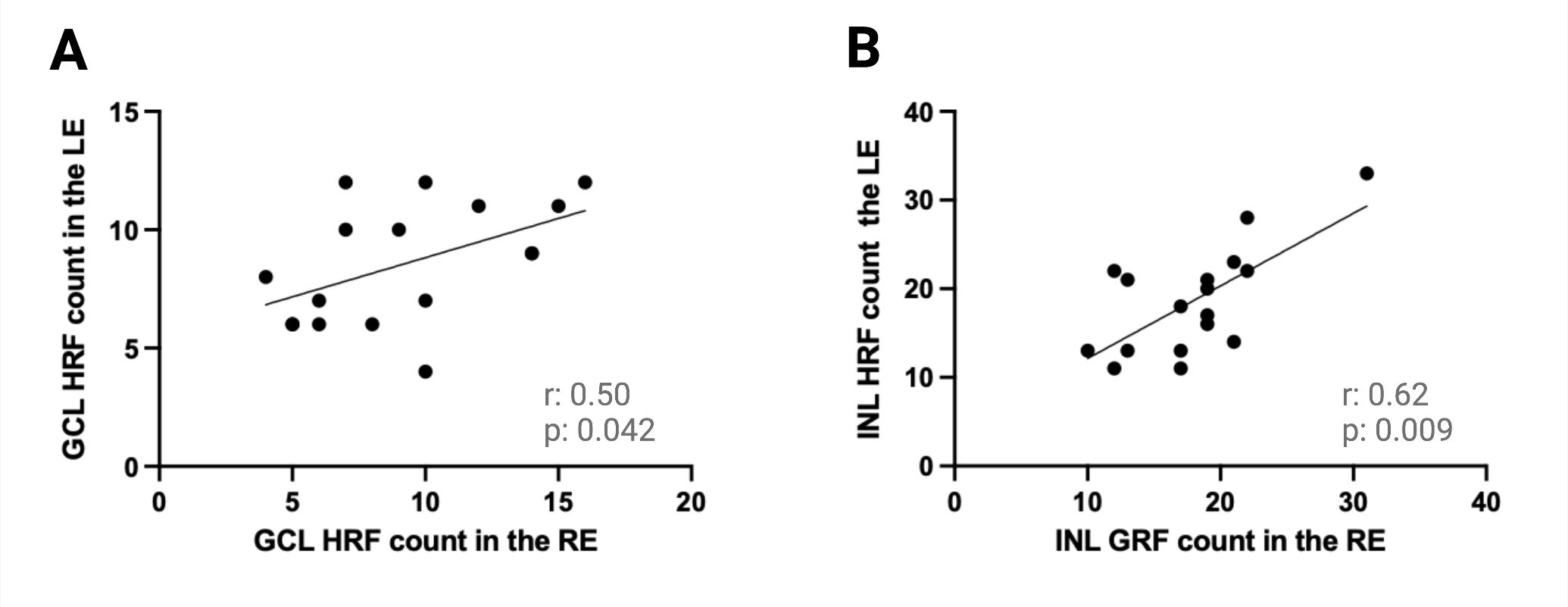

Supplement: Supplementary Figure 1 — Spearman Correlation of GCL HRF count between the two eyes (A) and of INL HRF count between the two eyes (B). [file Image_1.jpeg]
