# Supplementary material for: Hyper-Reflecting Foci in Multiple Sclerosis Retina Associate With Macrophage/Microglia-Derived Cytokines in Cerebrospinal Fluid
Source: Front Immunol. 2022 May 19;13:852183. doi: 10.3389/fimmu.2022.852183 (PMC9160385; doi:10.3389/fimmu.2022.852183)
Supplement: Supplementary Table 1 — Spearman Correlation between GCL HRF count and CSF cytokine concentrations in RRMS. [file Table_1.pdf]

	r-value	p-value
IL1-Ra cont	***-0.6714***	*0.0016* **
IL-9	***-0.6091***	***0.0056*** **
IL-15	***-0.6263***	***0.0041*** **
Eotaxin	0.1207	0.6226 ns
G-CSF	***-0.5099***	***0.0257*** *
IFN-γ	***-0.4894***	***0.0334*** *
MIP-1a	0.09123	0.7103 ns
MIP-1b	-0.3119	0.1936 ns
PDGF-BB	-0.2789	0.2476 ns
RANTES	-0.1231	0.6155 ns
VEGF	-0.326	0.1732 ns
APRIL	-0.03009	0.9027 ns
BAFF	-0.1463	0.5502 ns
sCD30	-0.02115	0.9315 ns
sCD163	-0.04493	0.8551 ns
sIL-6Rb	-0.02555	0.9173 ns
IFN-β	0.1692	0.4887 ns
sIL-6Ra	-0.03172	0.8974 ns
IL-10	0.08634	0.7252 ns
IL-11	0.1412	0.5643 ns
IL-19	0.1276	0.6027 ns
IL-20	0.1932	0.4281 ns
IL-22	-0.001767	0.9943 ns

	r-value	p-value
IL-26	0.1921	0.4308 ns
IL-27	0.2963	0.218 ns
IL-32	-0.2512	0.2995 ns
IL-34	-0.2226	0.3596 ns
IL-35	0.1043	0.6709 ns
LIGHT TNFSF1	-0.1709	0.4841 ns
Osteocalcina	-0.1383	0.5722 ns
Osteopontina	-0.2969	0.217 ns
Pentraxin-3	-0.1315	0.5917 ns
sTNF-R1	-0.1639	0.5026 ns
sTNF-R2	0.07577	0.7579 ns
TSLP	0.008853	0.9713 ns
TNFSF12	-0.3119	0.1936 ns
CCL-21	-0.3656	0.1237 ns
CXCL-13	0.3841	0.1044 ns
CCL-27	0.1841	0.4505 ns
CXCL-25	0.0736	0.7646 ns
CCL-24	-0.1216	0.62 ns
CCL-26	-0.06217	0.8004 ns
CX3CL-1	-0.2123	0.3828 ns
CXCL-6	-0.1151	0.6388 ns
GM-CSF	-0.2217	0.3617 ns
CXCL-1	-0.1076	0.6611 ns

	r-value	p-value
CXCL-2	-0.2865	0.2343 ns
CCL-1	-0.0511	0.8354 ns
IL-2	0.07607	0.7569 ns
IL-4	0.1154	0.638 ns
IL-6	-0.1172	0.6327 ns
IL-8	-0.03789	0.8776 ns
IL-16	-0.07753	0.7524 ns
CXCL-10	0.2626	0.2775 ns
CXCL-11	0.3295	0.1683 ns
CCL-2	-0.2423	0.3176 ns
CCL-8	-0.0004407	0.9986 ns
CCL-7	-0.07804	0.7508 ns
CCL-13	-0.05685	0.8172 ns
CCL-22	0.2626	0.2775 ns
MIF	-0.1225	0.6174 ns
CXCL-9	0.3101	0.1963 ns
CCL-15	-0.3004	0.2114 ns
CCL-19	-0.2211	0.3629 ns
CCL-23	-0.06079	0.8047 ns
CXCL-16	-0.06784	0.7826 ns
CXCL-12	-0.05727	0.8159 ns
CCL-25	0.0008811	0.9971 ns
TNF-α	0.2186	0.3686 ns
