# Supplementary material for: Hyper-Reflecting Foci in Multiple Sclerosis Retina Associate With Macrophage/Microglia-Derived Cytokines in Cerebrospinal Fluid
Source: Front Immunol. 2022 May 19;13:852183. doi: 10.3389/fimmu.2022.852183 (PMC9160385; doi:10.3389/fimmu.2022.852183)
Supplement: Supplementary Table 2 — Spearman Correlation between INL HRF count and CSF cytokine concentrations in RRMS. [file Table_2.pdf]

	r-value	p-value
IL1-Ra cont	-0.183	0.4533 ns
IL-9	-0.1813	0.4575 ns
IL-15	-0.1206	0.6229 ns
Eotaxin	0.09767	0.6908 ns
G-CSF	-0.07967	0.7458 ns
IFN-γ	-0.159	0.5157 ns
MIP-1a	0.3917	0.0972 ns
MIP-1b	-0.0396	0.8721 ns
PDGF-BB	-0.08918	0.7166 ns
RANTES	-0.009696	0.9686 ns
VEGF	0.1126	0.6462 ns
APRIL	-0.1498	0.5405 ns
BAFF	-0.1918	0.4315 ns
sCD30	0.03608	0.8834 ns
sCD163	0.1443	0.5556 ns
sIL-6Rb	0.2552	0.2917 ns
IFN-β	-0.3423	0.1514 ns
sIL-6Ra	0.337	0.1583 ns
IL-10	-0.1021	0.6776 ns
IL-11	0.08987	0.7145 ns
IL-19	-0.2734	0.2575 ns
IL-20	-0.1784	0.4649 ns
IL-22	***-0.4693***	***0.0426*** *

	r-value	p-value
IL-26	-0.3291	0.1689 ns
IL-27	-0.02818	0.9088 ns
IL-32	-0.06173	0.8018 ns
IL-34	***-0.5073***	***0.0266*** *
IL-35	***-0.4672***	***0.0437*** *
LIGHT TNFSF1	0.3819	0.1067 ns
Osteocalcina	-0.03696	0.8806 ns
Osteopontina	0.04487	0.8553 ns
Pentraxin-3	-0.02863	0.9074 ns
sTNF-R1	0.0176	0.943 ns
sTNF-R2	0.2235	0.3577 ns
TSLP	-0.03227	0.8957 ns
TNFSF12	0.08975	0.7148 ns
CCL-21	0.05983	0.8078 ns
CXCL-13	***0.498***	***0.03*** *
CCL-27	-0.1117	0.6488 ns
CXCL-25	-0.1461	0.5506 ns
CCL-24	0.01848	0.9402 ns
CCL-26	-0.2642	0.2744 ns
CX3CL-1	0.09503	0.6988 ns
CXCL-6	-0.1683	0.491 ns
GM-CSF	0.132	0.59 ns
CXCL-1	0.03831	0.8763 ns

	r-value	p-value
CXCL-2	***-0.5287***	***0.02*** *
CCL-1	-0.1214	0.6205 ns
IL-2	-0.2741	0.2561 ns
IL-4	-0.1452	0.5532 ns
IL-6	0.07042	0.7745 ns
IL-8	0.2824	0.2413 ns
IL-16	-0.1645	0.5009 ns
CXCL-10	***0.4716***	***0.0415*** *
CXCL-11	0.4355	0.0623 ns
CCL-2	0.00264	0.9914 ns
CCL-8	0.4115	0.08 ns
CCL-7	-0.2607	0.2811 ns
CCL-13	0.03081	0.9004 ns
CCL-22	0.4479	0.0545 ns
MIF	0.09415	0.7014 ns
CXCL-9	0.2173	0.3714 ns
CCL-15	0.01232	0.9601 ns
CCL-19	0.1681	0.4916 ns
CCL-23	-0.06687	0.7856 ns
CXCL-16	0.09415	0.7014 ns
CXCL-12	0.2657	0.2715 ns
CCL-25	-0.2349	0.333 ns
TNF-α	-0.02245	0.9273 ns
